# Feedback from Horizontal Cells to Cones Mediates Color Induction and May Facilitate Color Constancy in Rainbow Trout

**DOI:** 10.1371/journal.pone.0066216

**Published:** 2013-06-04

**Authors:** Shai Sabbah, Changhai Zhu, Mark A. W. Hornsby, Maarten Kamermans, Craig W. Hawryshyn

**Affiliations:** 1 Department of Biology, Queen's University, Kingston, Ontario, Canada; 2 Research Unit Retinal Signal Processing, The Netherlands Institute for Neuroscience, Amsterdam, The Netherlands; 3 Department of Neurogenetics, Academic Medical Center, University of Amsterdam, Amsterdam, The Netherlands; 4 Center for Neuroscience Studies, Queen's University, Kingston, Ontario, Canada; University of Oldenburg, Germany

## Abstract

Color vision is most beneficial when the visual system is color constant and can correct the excitations of photoreceptors for differences in environmental irradiance. A phenomenon related to color constancy is color induction, where the color of an object shifts away from the color of its surroundings. These two phenomena depend on chromatic spatial integration, which was suggested to originate at the feedback synapse from horizontal cells (HC) to cones. However, the exact retinal site was never determined. Using the electroretinogram and compound action potential recordings, we estimated the spectral sensitivity of the photoresponse of cones, the output of cones, and the optic nerve in rainbow trout. Recordings were performed before and following pharmacological inhibition of HC-cone feedback, and were repeated under two colored backgrounds to estimate the efficiency of color induction. No color induction could be detected in the photoresponse of cones. However, the efficiency of color induction in the cone output and optic nerve was substantial, with the efficiency in the optic nerve being significantly higher than in the cone output. We found that the efficiency of color induction in the cone output and optic nerve decreased significantly with the inhibition of HC-cone feedback. Therefore, our findings suggest not only that color induction originates as a result of HC-cone feedback, but also that this effect of HC-cone feedback is further amplified at downstream retinal elements, possibly through feedback mechanisms at the inner plexiform layer. This study provides evidence for an important role of HC-cone feedback in mediating color induction, and therefore, likely also in mediating color constancy.

## Introduction

Color vision, the ability to distinguish equally bright stimuli based on differences in spectral content, serves many animals in foraging, detecting predators, and communicating. It is therefore likely that color vision is a strongly selected character and important to investigate from behavioral, molecular and physiological perspectives. However, color vision would be most beneficial if the visual system can correct the excitations of the various photoreceptors by compensating for differences in environmental irradiance. To achieve that, the visual system first should decompose the spectral radiance arriving at the eye from an object into the spectral reflectance of the object, and the spectral irradiance in the environment. Such correction of photoreceptor excitations for differences in environmental irradiance would result in color constancy - the ability of the visual system to perceive colors as constant, despite considerable changes in the spectral composition of the illuminant.

A phenomenon related to color constancy is simultaneous color contrast or color induction - the perception that the color of an object shifts to the complementary color of its background. Both phenomena seem to originate, at least in part, from the same mechanism - chromatic spatial integration [1]–[5]. This common feature is intimately linked to the center-surround organization of receptive fields in the visual system, which was suggested to enhance luminance [6] and color [4], [7]–[9] contrast. Chromatic spatial integration occurs for the first time at the synapse between photoreceptors and horizontal cells (HC). It was suggested that, at least partly, color constancy and color induction originate there [4], [5]. However, this was never tested directly.

Center-surround organization is an elementary attribute of receptive fields in visual systems. It originates, at least in part, by HCs [10], [11], and is conserved downstream, through bipolar cells to ganglion cells, the output neurons of the retina [12]–[14]. The feedback synapse from HCs to cones, the first neural feedback loop in the visual system, was suggested to evoke the surround response in bipolar and ganglion cells [10], [11]. This feedback modulates the Ca^2+^ current of cones [15]; hyperpolarization of HCs shifts the Ca^2+^ current of cones to more negative potentials, leading to an increase of Ca^2+^-influx and an increase in glutamate release by the cones. The mechanism by which HC-cone feedback modulates Ca^2+^ current is yet controversial [16]. The modulation of cone Ca^2+^ current was suggested to result from ephaptic modulation of the cone pedicle membrane potential generated by currents flowing through hemichannels in HC dendrites [6], [17], [18], or from changes in proton levels at the synaptic cleft [19], [20].

The feedback from HCs to cones can be effectively inhibited by submillimolar concentrations of cobalt (Co^2+^) [11], [21], [22]; this inhibition is associated with a shift of the Ca^2+^ current of cones to positive potentials [17]. The cobalt-induced inhibition of HC-cone feedback was shown to substantially reduce the response and dimension of the receptive-field surrounds of ganglion cells and some bipolar and amacrine cells [11]. Additionally, recent intracellular recordings from HCs revealed that spectral opponent responses in HCs diminish when HC-cone feedback is impaired (in mutants lacking connexin hemichannels) [6]. Therefore, inhibition of the HC-cone feedback may interfere with the processing of chromatic information in the retina.

In this study, we tested whether color induction originates at the feedback synapse between HCs and cones in rainbow trout (*Oncorhynchus mykiss*). We estimated the spectral sensitivity of the photoresponse of cones (ERG a-wave) and the output of cones (ERG b-wave), and the spectral sensitivity of the output of the retina (compound action potential [CAP] of the optic nerve). Previous studies have used whole-cell patch clamp or sharp electrode recordings from individual cones and horizontal cells to dissect the mechanism underlying the HC-cone feedback. For example, Kraaij et al. [23] have measured the spectral sensitivity of cones and the feedback signal, and Klaassen et al. [6] have quantified the hemichannel currents in horizontal cells and the inward current in cones which is due to HC-cone feedback. Unlike whole-cell recordings, ERG and CAP measured from the optic nerve account for gross potentials, representing complex summations of output signals of different photoreceptor classes and bipolar cell types (ERG) and many types of ganglion cells (CAP). As such, ERG and CAP signals cannot provide high-resolution description of a mechanism as complex as HC-cone feedback. However, both ERG and CAP of the optic nerve are efficient ways to determine how the overall spectral sensitivity across diverse types of photoreceptors and retinal neurons changes in response to background modulation. Recordings were performed before and following inhibition of HC-cone feedback, and were repeated under two different background illumination conditions to estimate the efficiency of color induction. In our experiments, only the spectrum of the surrounding background was changed, while the spectrum of the stimulus was kept fixed. Using this setup, the cone photoreceptors are equally stimulated by a central light stimulus; therefore, the spectral sensitivity of the photoresponse of cones would not be expected to vary under different spectral surrounds. Conversely, the output of cones, modulated by feedback from HCs, depends on the surround spectrum; therefore, the spectral sensitivity of the output of cones would be expected to vary under different spectral surrounds, with sensitivity increasing at wavelengths strongly deviating from the wavelength of the background illumination. This behavior is characteristic for simultaneous color contrast [4].

We hypothesized that if HC-cone feedback mediates color induction, then (i) the efficiency of color induction in the photoresponse of cones would be lower than that in the output of cones, and (ii) the efficiency of color induction in the output of cones, but not in the photoresponse of cones, would decrease under cobalt-induced inhibition of HC-cone feedback. Additionally, we were interested in how the efficiency of color induction varies across retinal processing stages. We hypothesized that if the efficiency of color induction is amplified toward downstream retinal elements, then the efficiency of color induction would be lowest in the photoresponse of cones, higher in the output of cones, and highest at the optic nerve.

We found that the efficiency of color induction was lowest in the photoresponse of cones, higher in the output of cones, and highest at the optic nerve. Additionally, the efficiency of color induction in the output of cones and optic nerve, but not in the photoresponse of cones, decreased with the inhibition of HC-cone feedback. These findings suggest not only that color induction originates as a result of feedback from HCs to cones, but also that this effect of HC-cone feedback on the efficiency of color induction is conserved and further amplified between the output of cones and the optic nerve.

## Methods

### Ethics Statement

All experimental and animal care procedures were approved by Queen's University Animal Care Committee, under the auspices of the Canadian Council for Animal Care.

### Fish care and holding conditions

Parr (*n* = 53; body mass = 15±4 g; standard length = 9.7±1 cm) rainbow trout (*Onchorynchus mykiss*; Rainbow Springs Trout Hatchery, Thamesford, ON, Canada) were held in our aquatic facility under a 12 h∶12 h light-dark photoperiod. Facility lighting comprised of full spectrum fluorescent lamps (UV-Blue actinic and BlueMax lamps; Full Spectrum Solutions, Jackson, MI, USA). Fish were held at a temperature of 15±1°C and were fed trout pellets once daily (Martin Mills, Elmira, ON, Canada).

### Fish preparation

To estimate the spectral sensitivity of the photoresponse and output of cones, we recorded electroretinograms (ERG) from the cornea of the fish. To estimate the spectral sensitivity of the output of the retina, we recorded compound action potentials (CAP) from the optic nerve of the fish. Prior to ERG and CAP recordings, fish were immersed in a solution of 125 mg L^−1^ tricaine methanesulfonate (MS-222) until they reached stage III anaesthesia [24]. A general anaesthetic (metomidate hydrochloride; 0.3 mg g^−1^ body mass; Maranil; Syndel Laboratories, Qualicum Beach, BC, Canada) and an immobilizing agent (pancuronium bromide; 0.05 mg g^−1^ body mass; Conier Chem and Pharma, Chongqing, China) were injected subcutaneously. Test fish were placed in a holding cradle inside a Faraday cage and irrigated with aerated fresh water (temperature = 15±1°C, flow rate = 0.2 L min^−1^).

### ERG and CAP experimental apparatus

The optical system and recording apparatus have been described in detail elsewhere [25]–[27]. Two background channels using 250 W halogen lamps (24 V ELC, Eiko, Kansas City, KS, USA) provided constant background illumination to light adapt the eye. A bifurcated optical fiber (fused silica, numerical aperture, NA = 0.22; Fiberoptic Systems, Simi Valley, CA, USA) guided light from the two background channels to the electrophysiology rig. The intensity and spectral composition of background illumination were manipulated using interference cutoff filters and neutral density filters (Corion, Franklin, MA, USA). The stimulus channel used a 300 W xenon arc lamp and monochromator (Newport Oriel, Irvine, CA, USA; 300 W bulb, Ushio, Cypress, CA, USA). The intensity and duration of the stimulus were manipulated using a 0–3 optical density neutral density wedge (fused silica; Melles-Griot, Rochester, NY, USA) and an electronic shutter (UniBlitz D122 Shutter, Vincent Associates, Rochester, NY, USA). An optical fiber (fused silica; NA = 0.55; Fiberoptic Systems) guided light from the stimulus channel to the electrophysiology rig. The background and stimulus optical fibers were fit to a beam splitter to produce a stimulus beam (diameter 0.5 cm at the plane of the fish eye) contained within the background beam (diameter 1 cm). Both the stimulus and background were presented as spots.

### ERG electrode configuration

For ERG recordings, a glass electrode (1.5 mm outer diameter, 1 mm inner diameter, borosilicate glass; World Precision Instruments, Sarasota, FL, USA) pulled to a tip diameter of 80 µm (P-97 Flaming/Brown Micropipette puller; Sutter Instruments, Novato, CA, USA) was loaded with saline (0.684 M sodium chloride) and inserted into a saline-filled chlorided AgCl half-cell (A-M systems, Sequim, WA, USA). The electrode tip was placed on the dorsal-nasal corneal surface of the right eye. A ground electrode was placed on the caudal fin and a Teflon-coated chlorided-silver reference electrode (0.5 mm, A-M Systems, Carlsborg, WA, USA) was placed on the head of the fish.

### CAP electrode configuration

Prior to CAP recordings, the dermis and bone over the left optic tectum were removed using a surgical drill. Thereafter, a Teflon-coated chlorided-silver electrode (0.5 mm, A-M Systems) was inserted into the lumen of the optic nerve [28], [29], a ground electrode was placed on the caudal fin, and a reference electrode (Teflon-coated chlorided-silver electrode) was placed on the fish skull. To ensure proper placement of the recording electrode, the shape and latency characteristics of the observed waveform were compared for different electrode penetrations and could easily be distinguished from the response of the optic tectum [25].

### ERG and CAP recording procedure

Recordings commenced at least one hour after the onset of the light phase and completed before the onset of the dark phase to eliminate any circadian rhythm effects [30]. The duration of the light stimulus was 500 ms with an interstimulus interval of 5 s. The recorded signal was filtered (10 Hz high pass, 100 Hz low pass) and amplified (BMA-200, CWE Incorporated, Ardmore, PA, USA). This amplified signal was then analyzed with a 16-bit A/D data acquisition system (Micro 1401; Cambridge Electronic Design, Cambridge, UK) and Signal 4.0 software. Spectral sensitivity was measured in 10 nm increments, from 340 to 700 nm, in a staggered wavelength presentation to prevent adaptation to specific spectral regions. At each wavelength, the ERG or CAP response to eleven stimulus intensities (irradiance levels) was determined. A third order polynomial was fit to the response versus irradiance (RI) curve and the threshold irradiance that corresponded to a response criterion of 25 µV was interpolated [31]. Sensitivity was estimated as the reciprocal of this threshold irradiance. Log relative sensitivity curves were created by normalizing the log absolute sensitivity values to the maximum sensitivity across the spectrum [26].

### Sensitivity of the photoresponse of cones, the output of cones, and the optic nerve

A typical ERG waveform as evoked in response to step illumination consists of an initial corneal negative response (a-wave) representing the response of photoreceptors to the onset of light (hereafter ‘cone photoresponse’), followed by a corneal positive response (b-wave) representing the response of ON bipolar cells to the onset of light. This response can be considered as the output of cones since the ERG b-wave hardly depends on inner retinal processing [32], [33], but does depend on lateral inhibition by horizontal cells [34], [35]. Accordingly, the cone output was estimated as the amplitude of the b-wave, whereas the cone photoresponse alone was estimated as the amplitude of the a-wave. However, due to the strong depolarization of ON bipolar cells, photoreceptor response (a-wave) was typically masked. To isolate the cone photoresponse, sodium-L-aspartate (ASP) was injected into the ocular media of fish (10 µl of 125 mM ASP in Ringer's solution [23], resulting in an estimated final ocular concentration of 8.62 mM; average vitreous volume 145 µl). ASP is routinely used to isolate photoreceptor responses in ERG [36]–[39]. ASP is a substrate of the glutamate transporter in photoreceptors that competitively inhibits glutamate uptake [40]. Therefore, ASP induces an increase in glutamate at the synapse, and a reduction in the light-evoked input to post-receptoral elements. The excess of glutamate at the synapse may open horizontal-cell (HC) cationic channels with a depolarized reversal potential, and maintain the HCs at a relatively depolarized potential [41]. This might render the effect of modulation of endogenous transmitter release by light stimuli on the membrane potential of HCs limited, and also might reduce the feedback from HCs to cones. To ensure constant effect of ASP throughout the experiments, ERG recordings commenced at least 20 minutes following the ASP injection and were completed within 250 minutes (typically within 150 minutes) following the injection (S. Sabbah, F. E. Hauser, and C. W. Hawryshyn, unpublished data). Note that, the high ASP concentration (125 mM) would make the Ringer's solution hyper-osmotic. Increases in osmolarity tend to decrease the ERG b-wave, but not to affect the ERG a-wave [42]. In this study, ASP was used only when assessing the photoresponse of cones (ERG a-wave). Thus, the ASP-induced increase in osmolarity likely had little effect (if any) on our cone photoresponse estimates. Moreover, the decrease in ERG waves is likely to be similar across the spectrum. Such a decrease would change the absolute spectral sensitivity, but not the relative spectral sensitivity that was used in all our analyses. Thus, the effect of variation in intraocular osmolarity on our results is likely negligible.

A typical waveform of CAP from the optic nerve as evoked in response to step illumination consists of an initial depolarization phase representing the response of ON ganglion cells to the onset of light (ON response), followed by another depolarization phase representing the response of OFF ganglion cells to the offset of light (OFF response). Accordingly, the optic nerve response to the onset of light was estimated as the amplitude of the ON response.

### Background illumination conditions

A background light beam adapted the fish eye 30 minutes prior to the measurement, as well as during the measurement of spectral sensitivity. Measurements were conducted under two different background illumination conditions: (i) a broad-spectrum background condition (hereafter ‘Natural background’), simulating the fish natural photic environment by accurately reproducing the sideward irradiance measured at a depth of 3 m at Lake Cowichan, Vancouver Island, BC, Canada [43]; and (ii) a background condition designed to dampen the sensitivity of the LWS cone mechanism (hereafter ‘LW adaptation background’). The number of photons collected by the various cone pigment classes under the two backgrounds was estimated using a quantum catch model: 
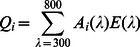
(1)


where *Q_i_* denotes the quantum catch of cone pigment *i*, *A*_i_(*λ*) denotes the spectral absorbance of cone pigment *i*, and *E*(*λ*) denotes the spectral photon irradiance of the background light. Absorbance spectra were generated for the four cone pigments reported in rainbow trout using the absorbance templates of Govardovskii [44]; the λ_max_ of visual pigments with an A_1_ chromophore were: UVS (365 nm), SWS (434 nm), MWS (531 nm), and LWS (576 nm) [45], [46]. We combined absorbance spectra for visual pigments with an A_1_ and A_2_ chromophore. The proportion of the A_2_ state was presented using a fraction parameter, *a* (0 ≤ *a* ≤ 1) and therefore, the absorbance spectrum of a given visual pigment exhibiting an A_2_ proportion of *a* was calculated as: 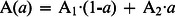
(2)


The λ_max_ of each visual pigment exhibits a defined wavelength shift as the A_2_ proportion changes [47]. This latter shift and the transmission of the lens, measured following Sabbah et al. [27], were taken into account when generating the absorbance spectra.

The irradiance provided under the various background conditions was measured at the plane of the fish eye using a spectroradiometer (QE65000; Ocean Optics, Dunedin, FL, USA) connected to an optical fiber (QP600-2-UV/VIS; Ocean Optics) that was fitted with a cosine corrector (CC-3-UV; Ocean Optics). The spectroradiometer setup was calibrated for absolute irradiance using a NIST (National Institute of Standards and Technology, Gaithersberg, MD, USA) calibrated Halogen-Deuterium dual light source (200–1000 nm, DH-2000-CAL; Ocean Optics) [48].

### Cobalt-induced inhibition of HC-cone feedback

Cobalt chloride (10 µl of 4.26 mM cobalt in Ringer's solution, resulting in an estimated final ocular concentration of 294 µM; average vitreous volume 145 µl) was injected intraocularly to inhibit HC-cone feedback. These injection volume and final ocular concentration were previously used to inhibit the HC-cone feedback in the same species and life stage, rainbow trout juveniles [24]. Additionally, the ocular cobalt concentration used (294 µM) falls within the concentration range of cobalt (100–500 µM) that has been shown to inhibit HC-cone feedback without interfering with feedforward synaptic transmission from cones to HCs [11], [21], [22].

### Estimation of the efficiency of color induction

Visual inspection of sensitivity spectra pairs acquired under the two background illumination conditions is indispensable when attempting to study the efficiency and characteristics of color induction. Nonetheless, to reduce the spectral data and to facilitate comparison of the efficiency of color induction across retinal processing stages and pharmacological manipulations, we employed a simple index. We used the root mean square error (RMSE) between the spectral sensitivity under the two backgrounds:
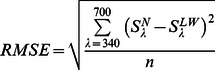
(3)


where 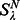 and 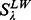 denote the sensitivity at wavelength *λ* as measured under the Natural and LW adaptation backgrounds, respectively; *n* denotes the number of data points across the spectrum (*n* = 37 for the spectral range 340 – 700 nm with a 10 nm interval). Note that in several cases, sensitivity measurements under the two backgrounds could not be obtained from the same individual. Therefore, RMSE was calculated between the sensitivity under the two backgrounds for all possible spectra pairs. For example, assuming sensitivity spectra from 4 individuals under Natural background and from 5 individuals under the LW adaptation background, RMSE was calculated between all 20 possible spectra pairs.

Moreover, to allow comparison of RMSE values across different pharmacological manipulations and retinal processing stages, prior to RMSE calculation, we standardized the sensitivity difference between the spectral sensitivity under the two backgrounds. This was achieved by minimizing the sensitivity difference between the sensitivity spectrum measured under the Natural background and that measured under the LW adaptation background, i.e., shifting the latter spectrum along the sensitivity (ordinate) axis. Importantly, the use of the RMSE as an index for the efficiency of color induction is appropriate only if the variation in sensitivity across the spectrum (between adjacent wavelengths) is similar for sensitivity measured at different retinal processing stages and when using different pharmacological manipulations. To test this assumption, the standard deviation across the spectrum for each sensitivity measurement was divided by the peak-to-peak amplitude of the spectrum. By doing so, we accounted only for variation between adjacent wavelengths (the wiggliness of the spectrum) while normalizing for the amplitude of the spectrum. We will show that the variation between adjacent wavelengths is indeed similar for sensitivity measured using different pharmacological manipulations, and across two (out of three) processing stages.

To gain further insight into the efficiency of color induction, we calculated the ratio between long-wavelength and ultraviolet sensitivity, for the Natural (*R_N_*) and LW adaptation (*R_LW_*) backgrounds; and we compared between *R_N_* and *R_LW_*. These sensitivity ratios were calculated as:
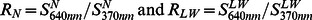
(4)


where 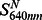 and 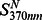 denote the sensitivity at 640 nm and 370 nm as measured under the Natural background, whereas 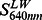 and 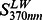 denote the sensitivity at 640 nm and 370 nm as measured under the LW adaptation background. Note that the wavelengths 640 nm and 370 nm correspond well to the wavelengths of maximum sensitivity of the LWS and UVS cones when the cone mechanisms interact with one another, as measured from an intact retina [49], [50]. Thus, the sensitivity at the wavelengths 640 nm and 370 nm was used for calculating *R_N_* and *R_LW_* in the cone output and optic nerve. In contrast, sensitivity of the cone photoresponse does not account for cone interaction, and therefore does not show a shift in the maximum sensitivity of the LWS toward longer wavelengths [51]. Therefore, *R_N_* and *R_LW_* in the photoresponse of cones were calculated using the wavelengths 580 nm and 370 nm.

### Statistical analysis

RMSE values, serving as an index for the efficiency of color induction, did not follow normal distribution (Kolmogorov-Smirnov test) and their variance differed across pharmacological manipulations and retinal processing stages (Leven's test). Therefore, to examine the effect of cobalt-induced inhibition of HC-cone feedback on RMSE, as well as to examine how RMSE varies between retinal processing stages, we used a randomization test, with the difference between the means of RMSE values of any two treatments/processing stages as a test statistic. The observed test statistic was compared to the null distribution estimated from 10,000 replicates, where RMSE values were randomly permutated while maintaining the original sample sizes [52]. Additionally, non-parametric percentile-based bootstrapping (10,000 replicates) was used to estimate the 95% (two-tailed) confidence intervals around the mean of RMSE values for each treatment/processing stage [53]. Similar statistical procedures were employed for analyzing the normalized standard deviation data of sensitivity spectra (used to estimate the variation across sensitivity spectra), and for analyzing the *R_N_* and *R_LW_* data (used for studying the efficiency of color induction across retinal processing stages). Statistical analyses were performed using R 2.15.0 (The R Foundation for Statistical Computing).

## Results

**Figure 1:**
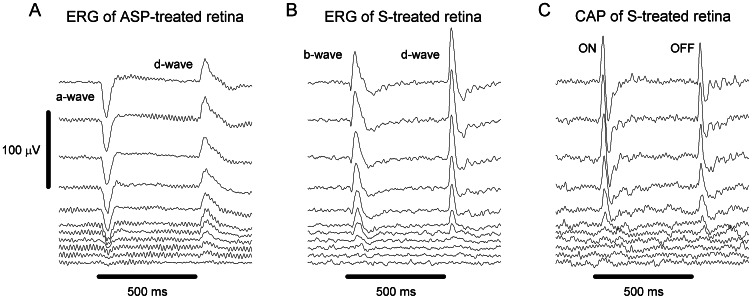
Waveforms for ERG of ASP-treated retina, and for ERG and CAP of saline-treated retina. (A) Waveforms of electroretinogram (ERG) of aspartate (ASP)-treated retina, (B) ERG of saline (S)-treated retina, and (C) compound action potential (CAP) of S-treated retina at increasing irradiance levels. Stimulus irradiance was incremented in 0.3 log unit steps. The ERG a-wave from ASP-treated retina was used to estimate the photoresponse of cones; the ERG b-wave from S-treated retina was used to estimate the light-evoked response of the output of cones; and the ON response of CAP from S-treated retina was used to estimate the light-evoked response of the optic nerve. Note that individual waveforms were vertically displaced for clarity of presentation. Horizontal thick lines indicate the time and duration of the light stimulus.

Spectral sensitivity was measured at different processing stages in the retina of rainbow trout. Sensitivity of the photoresponse of cones was estimated from the ERG a-wave of retina treated with aspartate (ASP) in saline (Ringer's solution); the sensitivity of the output of cones was estimated from the ERG b-wave of saline-treated retina; and the sensitivity of the optic nerve was estimated from the ON response of the compound action potential (CAP) of the optic nerve, projecting from saline-treated retina. See Figure 1 for ERG waveforms recorded from ASP-treated and saline-treated retina, as well as for CAP waveforms recorded from the optic nerve projecting from saline-treated retina.

**Figure 2:**
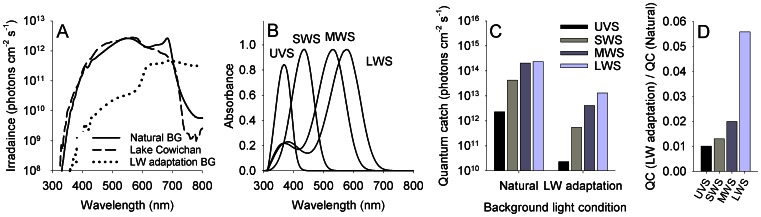
Background illumination conditions, and absorbance spectra and quantum catch of cone pigments in rainbow trout. (A) Two background illumination conditions were used, a broad-spectrum ‘Natural’ background and a long wavelength (LW) adaptation background. The broad-spectrum background simulated the fish natural photic environment by reproducing the sideward irradiance measured at a depth of 3 m at Lake Cowichan, Vancouver Island, BC, Canada (root mean square error = 0.38 log photons cm^−2^ s^−1^; Pearson *r* = 0.94). The LW adaptation background was designed to dampen the sensitivity of the LWS cone mechanism. (B) Absorbance spectra of the cone pigment classes in rainbow trout. Based on the range of photoreceptor sensitivity, absorbance spectra were constructed while assuming visual pigments are of A1 chromophore. (C) The number of photons collected by the various cone pigment classes under the background conditions used, as estimated using a quantum catch model. (D) The ratio between the quantum catch (QC) of pigments under the LW adaptation and Natural backgrounds was highest for LWS, indicating that LWS is the pigment being adapted most extensively under the LW adaptation compared to the Natural background.

Spectral sensitivity was measured under two different background illumination conditions. To evaluate the spectral sensitivity under natural photic conditions, measurements were conducted under a broad-spectrum background illumination that accurately reproduced the natural photic conditions encountered at the native environment of rainbow trout (‘Natural background’). Spectral sensitivity was also measured under background illumination that selectively adapted the LWS cones (‘long wavelength (LW)-adaptation background’). Figure 2 shows the spectral irradiance of the background conditions used, the absorbance spectra of cone pigments in rainbow trout, and the quantum catch for each cone pigment under both backgrounds.

### How does horizontal cell-cone feedback alter the efficiency of color induction?

**Figure 3:**
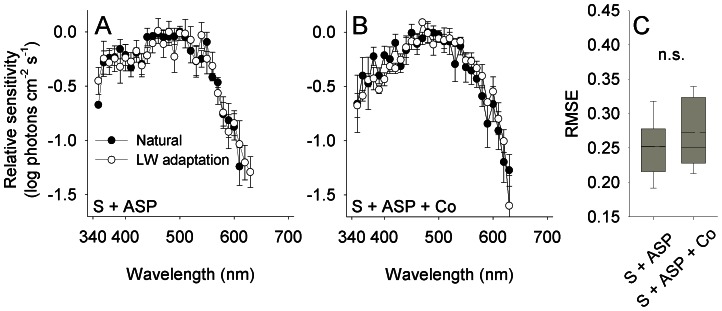
HC-cone feedback does not affect the efficiency of color induction in the photoresponse of cones. (A,B) Spectral sensitivity of the photoresponse of cones measured from retina treated with saline and aspartate (S + ASP), and from retina treated with saline, aspartate and cobalt (S + ASP + Co). A sensitivity peak in the ultraviolet region (ca. 370 nm) in the S+ASP-treated retina disappeared following the application of cobalt. Error bars, ±1 SEM. Sample size: S + ASP: Natural = 5, LW adaptation = 5; S + ASP + Co: Natural = 5, LW adaptation = 4. (C) The efficiency of color induction, as indicated by the root mean square error (RMSE) between the spectral sensitivity under the two backgrounds, did not vary significantly with the application of cobalt that inhibits HC-cone feedback (denoted n.s.; see text for statistics). Box: mean (dashed), median (solid), 25^th^ and 75^th^ percentiles; whiskers: 10^th^ and 90^th^ percentiles.

To study the effect of HC-cone feedback on the efficiency of color induction in the photoresponse of cones, spectral sensitivity of the photoresponse of cones was measured by ERG from retina treated with saline (and ASP) as well as from retina treated with saline and cobalt (and ASP) (Figure 3A,B). The efficiency of color induction was defined as the extent by which two sensitivity spectra, obtained under the two backgrounds, differed. We used the root mean square error (RMSE) between the two sensitivity spectra as an index for this difference. See *Methods* for detailed description of estimation of the efficiency of color induction.

The efficiency of color induction in the photoresponse of cones did not differ significantly between saline-treated and cobalt-treated retina (randomization test [RT], *P* = 0.199; confidence interval 2.5–97.5%–CI_saline_ = 0.234–0.271, CI_cobalt_ = 0.249–0.299, *n*_saline_ = 25, *n*_cobalt_ = 20) (Figure 3C). This indicates that cobalt did not affect spatial chromatic integration at the photoreceptors level. Note, however, that there might be a small overall change in spectral sensitivity following the inhibition of HC-cone feedback using cobalt (compare Figure 3A and B). This change, however, was independent of the color induction protocol and therefore not relevant to the present study.

**Figure 4:**
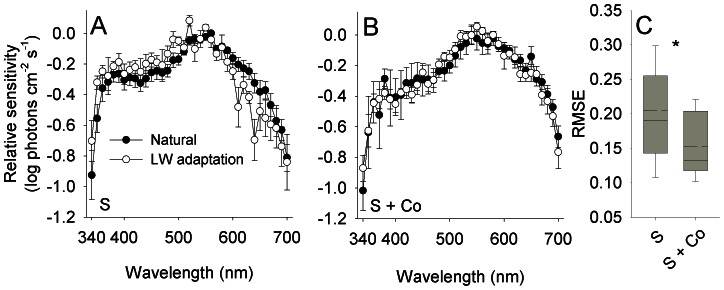
HC-cone feedback increases the efficiency of color induction in the output of cones. (A,B) Spectral sensitivity of the output of cones measured from saline-treated retina (S) and retina treated with saline and cobalt (S + Co). Error bars, ±1 SEM. Sample size: S: Natural = 5, LW adaptation = 5; S + Co: Natural = 4, LW adaptation = 5. (C) The efficiency of color induction, as indicated by the RMSE between the spectral sensitivity under the two backgrounds, decreased significantly with the application of cobalt (marked with an asterisk; see text for statistics). Box specifications as in Figure 3.

To study the effect of HC-cone feedback on the efficiency of color induction in the output of cones, spectral sensitivity of the output of cones was measured by ERG from retina treated with saline and from retina treated with saline and cobalt (Figure 4A-C). The efficiency of color induction decreased significantly with the application of cobalt (RT, *P* = 0.006; CI_saline_ = 0.178–0.232, CI_cobalt_ = 0.133–0.172; *n*_saline_ = 25, *n*_cobalt_ = 20) (Figure 4C). Therefore, HC-cone feedback significantly increased the efficiency of color induction in the output of cones.

**Figure 5:**
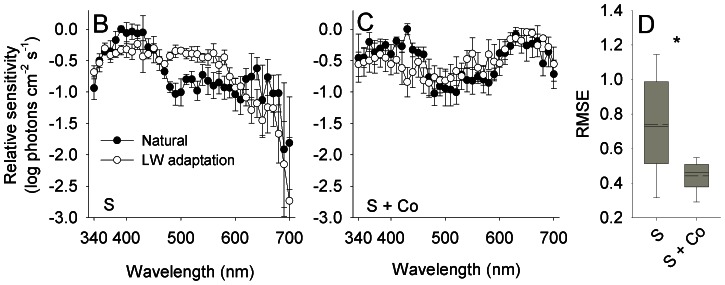
HC-cone feedback increases the efficiency of color induction at the optic nerve. (A,B) Spectral sensitivity of the optic nerve measured from saline-treated retina (S) and retina treated with saline and cobalt (S + Co). Application of cobalt induced a change in the shape of spectral sensitivity, increasing the sensitivity to long wavelengths (observed for sensitivity measured under both backgrounds). Error bars, ±1 SEM. Sample size: S: Natural = 5, LW adaptation = 5; S + Co: Natural = 5, LW adaptation = 5. (C) The efficiency of color induction, as indicated by the RMSE between the spectral sensitivity under the two backgrounds, decreased significantly with the application of cobalt (marked with an asterisk; see text for statistics). Box specifications as in Figure 3.

To study the effect of HC-cone feedback on the efficiency of color induction at the optic nerve, spectral sensitivity was measured from the optic nerve projecting from retina treated with saline and from retina treated with saline and cobalt (Figure 5A-C). The efficiency of color induction decreased significantly with the application of cobalt (RT, *P*<0.001; CI_saline_ = 0.626–0.847, CI_cobalt_ = 0.404–0.476; *n*_saline_ = 25, *n*_cobalt_ = 20) (Figure 5C). Therefore, HC-cone feedback significantly increased the efficiency of color induction at the optic nerve.

Note that comparison of RMSE values between saline-treated retina and cobalt-treated retina is meaningful only if the variation in sensitivity across the spectrum is similar for the two pharmacological manipulations. This requirement was met. The variation across the spectrum, estimated as the normalized standard deviation (see *Methods* for details), did not differ significantly between sensitivity measured from saline-treated retina and cobalt-treated retina (RT, cone photoresponse, *P* = 0.337; CI_saline_ = 0.237–0.264, CI_cobalt_ = 0.221–0.259; *n*_saline_ = 10, *n*_cobalt_ = 9; cone output, *P* = 0.508; CI_saline_ = 0.214–0.236, CI_cobalt_ = 0.205–0.234; *n*_saline_ = 10, *n*_cobalt_ = 9; optic nerve, *P* = 0.142; CI_saline_ = 0.218–0.254, CI_cobalt_ = 0.242–0.269; *n*_saline_ = 10, *n*_cobalt_ = 10). This demonstrates the applicability of the comparison of the efficiency of color induction (RMSE values) between the two pharmacological manipulations.

### How does color induction change across different retinal processing stages?

Recall that comparison of RMSE values between different processing stages is meaningful only if the variation in sensitivity across the spectrum is similar for the processing stages in concern (cone photoresponse vs. cone output vs. optic nerve). Specifically, the variation across the spectrum, measured from saline-treated retina and estimated as the normalized standard deviation, differed significantly between sensitivity of the cone photoresponse (CP) and cone output (CO) (RT, *P* = 0.017; CI_cp_ = 0.237–0.264, CI_co_ = 0.213–0.236; *n*_cp_ = 10, *n*_co_ = 10), but not between sensitivity of the output of cones and optic nerve (ON) (RT, *P* = 0.324; CI_co_ = 0.213–0.236, CI_ON_ = 0.218–0.254; *n*_co_ = 10, *n*_ON_ = 10). Therefore, a meaningful comparison of RMSE values was possible only between the sensitivity of the output of cones and optic nerve, as measured from saline-treated retina. The efficiency of color induction differed significantly between the cone output and the optic nerve as measured from saline-treated retina (RT, *P*<0.001; CI_co_ = 0.177–0.232, CI_ON_ = 0.630–0.846; *n*_co_ = 25, *n*_ON_ = 25). This finding suggests that the efficiency of color induction is being amplified by a factor of approximately 3.6 between the output of cones and optic nerve (average, RMSE_co_ = 0.205, RMSE_ON_ = 0.738).

**Figure 6:**
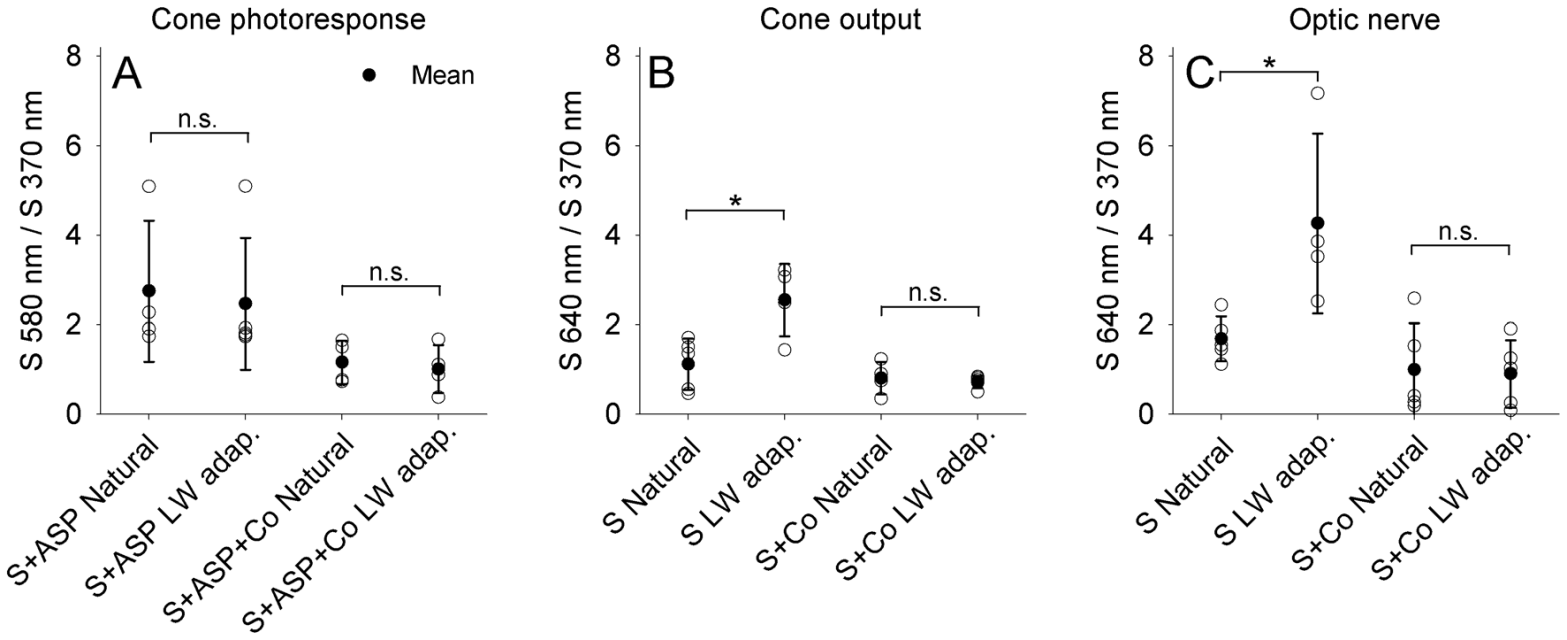
Efficiency of color induction varies between the photoresponse of cones, output of cones, and optic nerve. (A) In the photoresponse of cones, the sensitivity ratio between UV and long wavelengths (580 nm/370 nm) under the Natural background (*R_N_*) did not differ significantly from that under the LW adaptation background (*R_LW_*), for both retina treated with saline (S + ASP) and retina treated with saline, aspartate and cobalt (S + ASP + Co). This suggests minimal efficiency of color induction in the photoresponse of cones, regardless of whether HC-cone feedback is inhibited or not. (B) In the output of cones and (C) optic nerve, the sensitivity ratio between UV and long wavelengths (640 nm/370 nm) under the Natural background was significantly smaller than under the LW adaptation background, for saline-treated retina (S) but not for retina treated with saline and cobalt (S + Co). This suggests relatively high efficiency of color induction in the output of cones and optic nerve. This efficiency, however, decreased with the application of cobalt and became insignificant. Note that the relationship between the sensitivity ratio under the Natural and LW adaptation conditions is in agreement with the estimation of the quantum catches of cone pigments. Filled symbols, mean of either *R_N_* or *R_LW_*; open symbols, individual *R_N_* or *R_LW_* values; error bars, ±1 SD; n.s., non-significant; asterisk, significant difference (*P*<0.05). Sample size: cone photoresponse, S + ASP Natural = 4, S + ASP LW adaptation = 5, S + ASP + Co Natural = 4, S + ASP + Co LW adaptation = 5; cone output, S Natural = 5, S LW adaptation = 4, S + Co Natural = 4, S + Co LW adaptation = 5; optic nerve, S Natural = 5, S LW adaptation = 4, S + Co Natural = 5, S + Co LW adaptation = 5. See *Methods* for details of the choice of wavelengths used for the calculation of *R_N_* and *R_LW_*. Note that spectra missing sensitivity values at the wavelengths used for the calculation of *R_N_* and *R_LW_* (370 and 580 nm for cone photoresponse, and 370 and 640 nm for cone output and optic nerve) were omitted from analysis.

To complete our analysis of the variation in the efficiency of color induction across different retinal processing stages, and to substantiate further our findings regarding the effect of HC-cone feedback on color induction, we took a different approach. We calculated the ratio between long-wavelength and ultraviolet sensitivity, for the Natural (*R_N_*) and LW adaptation (*R_LW_*) backgrounds (see *Methods* for a detailed description of the calculation of sensitivity ratios). A non-significant difference between *R_N_* and *R_LW_* may suggest minimal color induction. In contrast, a significant difference between *R_N_* and *R_LW_* may suggest substantial color induction. See Table 1 for detailed statistics of the effect of background modulation on the ratio between long-wavelength and ultraviolet sensitivity, across retinal processing stages. For the photoresponse of cones, *R_N_* and *R_LW_* did not differ significantly, for both saline-treated retina and cobalt-treated retina (Figure 6A). This suggests minimal (or zero) efficiency of color induction in the photoresponse of cones, regardless of whether HC-cone feedback is inhibited or not. On the other hand, for the output of cones and optic nerve, *R_N_* was significantly smaller than *R_LW_* for saline-treated retina, but not for cobalt-treated retina (Figure 6B,C). This suggests relatively high efficiency of color induction in the output of cones and optic nerve. This efficiency, however, decreased with the application of cobalt and became insignificant; supporting our findings of the importance of HC-cone feedback in mediating color induction.

**Table 1:** Statistics of the effect of background illumination on the ratio between long-wavelength and ultraviolet sensitivity, across retinal processing stages.

Processing stage	Treatment	*P* *	CI Natural (*n*)	CI LW adaptation (*n*)
Cone photoresponse	saline	0.564	1.818–4.289 (4)	1.759–3.785 (5)
	cobalt	0.716	0.739–1.572 (4)	0.553–1.471 (4)
Cone output	saline	**0.033**	0.673–1.553 (5)	1.841–3.135 (4)
	cobalt	0.698	0.481–1.1088 (4)	0.601–0.811 (5)
Optic nerve	saline	**0.007**	1.328–2.093 (5)	2.851–6.249 (4)
	cobalt	0.882	0.259–1.907 (5)	0.333–1.511 (5)

* Significant effects (*P*<0.05) are marked in bold.

## Discussion

This study provides evidence for an important role of feedback from HCs to cones in shaping spectral sensitivity and mediating color induction, and therefore, likely also in mediating color constancy. Our results show that the efficiency of color induction in the output of cones and optic nerve decreased following inhibition of feedback from HCs to cones. These findings are consistent with the idea that HC-cone feedback is instrumental in the ability of the retina to achieve color constancy. Our results also indicate that the efficiency of color induction was not only conserved at the level of the optic nerve, but was also amplified further, possibly through a feedback loop at the inner plexiform layer.

### Effect of feedback from horizontal cells to cones on color induction

We hypothesized that if color induction results from feedback from HCs to cones, then (i) color induction in the photoresponse of cones would be absent, and (ii) the efficiency of color induction in the output of cones would decrease under cobalt-induced inhibition of HC-cone feedback. First, inhibition of HC-cone feedback was indeed observed to significantly decrease the efficiency of color induction in the output of cones, but not in the photoresponse of cones (Figures 3,4). This observation was based on the analysis of RMSE values between sensitivity spectra obtained under the two distinct backgrounds. Our finding regarding the photoresponse of cones is further supported by the non-significant difference between the sensitivity ratios *R_N_* and *R_LW_* for cobalt-treated retina (Figure 6A), which indicates no effect of inhibition of HC-cone feedback on the efficiency of color induction in the photoresponse of cones. Moreover, our finding regarding the sensitivity of the output of cones is further supported by the significant difference between *R_N_* and *R_LW_* for saline-treated retina, and the non-significant difference between *R_N_* and *R_LW_* for cobalt-treated retina (Figure 6B); these indicate a strong effect of inhibition of HC-cone feedback on the efficiency of color induction in the output of cones. Second, based on comparison of the *R_N_* and *R_LW_* sensitivity ratios for saline-treated retina, the efficiency of color induction in the output of cones was found to be substantial (significant difference between *R_N_* and *R_LW_*), while that in the photoresponse of cones was found to be minimal or zero (non-significant difference between *R_N_* and *R_LW_*). Taken together, these findings suggest that color induction originates at the synapse between photoreceptors and HCs, as a result of negative feedback from HCs to cone photoreceptors.

Moreover, the effect of HC-cone feedback on color induction was not restricted to the outer retina. In fact, our results showed that inhibition of HC-cone feedback significantly decreased the efficiency of color induction at the optic nerve (Figure 5). This finding regarding the sensitivity of the optic nerve was further supported by the significant difference between *R_N_* and *R_LW_* for saline-treated retina, and the non-significant difference between *R_N_* and *R_LW_* for cobalt-treated retina (Figure 6C); these indicate an effect of inhibition of HC-cone feedback on the efficiency of color induction at the optic nerve. That is, the effect of HC-cone feedback on color induction propagates throughout the retina, and can be detected at the optic nerve, the output of the retina.

Note that, cobalt may affect retinal physiology in ways other than inhibiting HC-cone feedback. For example, cobalt may inhibit the GABA-induced current in cones [54] and may also affect inner retinal GABAergic retinal circuits. Thus, it is possible that some of the observed effect of cobalt on spectral sensitivity has been mediated via processes other than the inhibition of HC-cone feedback. This highlights the importance of examination of the role of GABAergic circuits in color induction. Additionally, cobalt may also block calcium channels [21]. By blocking calcium channels in cones and reducing the feedforward strength from cones to horizontal cells, this would necessarily reduce HC-cone feedback, but would not directly affect the HC-to-cone synapse. Note, however, such a reduction in feedforward strength from cones to HCs occurs with 5–10 mM cobalt concentrations. In contrast, in this study, we have used a submillimolar cobalt concentration of 294 µM; similar submillimolar cobalt concentrations were repeatedly shown to inhibit HC-cone feedback without interfering with feedforward synaptic transmission from cones to HCs [11], [21], [22]. This supports our interpretation that the cobalt-induced reduction in the efficiency of color induction in the cone output resulted from inhibition of HC-cone feedback. However, it is possible that part of the cobalt-induced reduction in the efficiency of color induction observed at the optic nerve resulted from inhibition of calcium channels in inner retina neurons. Furthermore, modulation of calcium current in cones, and therefore, inhibition of HC-cone feedback, can also be achieved by buffering the pH (e.g., using HEPES) at the HC-to-cones synaptic cleft [16], [19], [20]. Thus, it would be beneficial to use HEPES to buffer the pH in the HC-to-cones synaptic cleft as a second means to demonstrate that color induction originates at the feedback synapse between HCs and cones.

### Variation in color induction across retinal processing stages

Apart from examining the effect of HC-cone feedback, we have also charted the variation in the efficiency of color induction throughout three retinal processing stages, i.e., in the cone photoresponse, cone output, and optic nerve. We found that the efficiency of color induction increased toward downstream retinal elements. As partially mentioned above, based on the comparison of the *R_N_* and *R_LW_* sensitivity ratios for saline-treated retina, the efficiency of color induction in the photoresponse of cones was absent (non-significant difference between *R_N_* and *R_LW_*), while that in the output of cones and optic nerve was found to be substantial (significant difference between *R_N_* and *R_LW_*) (Figure 6). Additionally, based on the analysis of RMSE values, the efficiency of color induction at the optic nerve was found to be significantly higher than in the output of cones. We already showed that the differences in the efficiency of color induction between the photoresponse and output of cones arise from HC-cone feedback, by which color induction originates at the synapse between the cone photoreceptors and HCs. However, our results also indicate amplification by a factor of 3.6 in the efficiency of color induction between the output of cones and optic nerve. The source of this amplification is currently unknown.

### Feedback from horizontal cells to cones contributes to color constancy

Our results suggest that color induction originates at the synapse between photoreceptors and HCs, as a result of HC-cone feedback. The ability of the retina to undergo color induction depends on chromatic spatial integration, which is also the basis for color constancy [1]–[5]. Consequently, color constancy and simultaneous color contrast or color induction are likely to originate as a result of HC-cone feedback. Chromatic spatial integration mechanisms require the ability to integrate the stimulus spatially as well as to control the gain, while accounting for interactions between cone classes [5]. These two requirements are satisfied for the first time at the level of the outer plexiform layer, where cones project to HCs, which in turn project back to cones. HCs integrate the stimulus spatially via their strong electrical coupling. HCs also feed back to cones by modulating the calcium current of cones; activation of this feedback mechanism leads to an increase in synaptic gain [4]. Moreover, HCs receive input from more than one cone type and feed back to more than one cone type [8], [55], [56], resulting in modulation of the output of cones based on the spectral content of the surround. Thus, the requirements to allow for chromatic spatial integration, and therefore, also for color constancy and color induction are fulfilled at the synapse between HCs to cones. Indeed, the cone-HC network was modeled, and was suggested to modulate the cone synaptic gains such that the ratios of cone outputs become almost invariant with the spectral composition of the global illumination, and therefore allow for color constancy and simultaneous color contrast [4].

Note that the basis of both phenomena, color induction and color constancy, is likely a single mechanism - chromatic spatial integration [1]–[5]. The two phenomena involve the adjustment of spectral sensitivity in response to modulation of the background (surround). Color constancy and simultaneous color contrast are almost instantaneous, and can be achieved in a few milliseconds [57]. Unfortunately, the experimental setup used in this study does not allow for evaluation of the time necessary to achieve the concerned adjustment in spectral sensitivity. Therefore, at present, we cannot be certain whether the adjustment observed in spectral sensitivity occurred instantaneously or over time. Nonetheless, regardless of the exact timing, our results demonstrate that the adjustment in spectral sensitivity in rainbow trout is mediated by negative feedback from HCs to cones.

Our results most probably can be generalized to fish and other lower vertebrates. However, can these results be generalized also to primates? And more specifically, how similar is the HC-cone feedback in fish and primates? The structure and physiology of the outer retina of fish and primates appear to differ in several aspects. Fish and primates might differ in the number of cone classes and in their peak sensitivities. For example, rainbow trout have four cone classes, whereas primates have only three cone classes. Additionally, the peak sensitivity of the M- and L-cones in primates differs by only 21 nm, whereas the peak sensitivity of the MWS and LWS in trout might differ by 46 to 83 nm, dependent on A_2_ chromophore proportion. Moreover, primates have only two cone-driven HC types; both types respond to all wavelengths with membrane hyperpolarization [56]. The response characteristics of HCs in rainbow trout are currently unknown. However, fish typically have two general types of cone-driven HCs. Monophasic HCs whose membrane hyperpolarizes to all spectral stimuli (non-spectral opponent HCs; as in primates), and multiphasic HCs whose membrane hyperpolarizes to some wavelengths and depolarizes to others (spectral opponent HCs) [8], [9], [55], [58]–[60]. Yet, HC-cone feedback in primates seems to modulate the cone calcium current [22], similarly to the feedback in goldfish, suggesting that a similar gain control mechanism is present in the synapse between HCs and cones. Additionally, the spectral sensitivity of the feedback in primates has not been characterized. However, HC connectivity together with the observation that HC-cone feedback in primates cannot be explained by the spectral sensitivity of a single cone class [22], suggest that the feedback signal is not spectrally opponent and shows spectral sensitivity broader than that of a single cone system, just like in fish [4], [23]. Moreover, the lack of spectral opponent HCs in primates can be accounted for by the largely overlapping L- and M-cone spectra [5], [61]. Therefore, despite several differences in the characteristics of the outer retina, we suggest that the basic mechanisms of outer retinal processing in fish and primates are highly similar, and that the differences in HC-cone feedback between fish and primates are merely qualitative. Nevertheless, caution is recommended if our results for rainbow trout are to be generalized to primates.

Color constancy is ubiquitous throughout the animal kingdom, and has been demonstrated in disparate taxa such as bees [62], fish [63], [64], and primates [65]. This indicates that the ability to hold colors constant, despite variation in the spectral composition of the illuminant, has a strong selective component, and was likely a driving factor in the development and retention of color vision among vertebrate and invertebrate lineages. This study suggests that the ability to perceive colors as constant depends on the feedback from HCs to cones.
